# Ophthalmic Manifestations of HIV Patients in a Rural Area of Western Maharashtra, India

**DOI:** 10.1155/2014/347638

**Published:** 2014-11-11

**Authors:** Pratik Y. Gogri, Somen L. Misra, Raghunandan N. Kothari, Akshay J. Bhandari, Hitesh V. Gidwani

**Affiliations:** ^1^Department of Ophthalmology, Rural Medical College and Pravara Rural Hospital of Pravara Institute of Medical Sciences, Loni, Ahmednagar, Maharashtra 413736, India; ^2^Department of Ophthalmology, Pravara Institute of Medical Sciences, Loni, Maharashtra 413736, India; ^3^Department of Ophthalmology, Smt. B. K. Shah Medical Institute & Research Centre, Vadodara, Gujarat 391750, India; ^4^Department of Medicine, UPMC Mercy, Pittsburgh, PA 15219, USA

## Abstract

*Introduction.* HIV/AIDS is one of twenty first century's biggest global challenges to mankind with protean manifestations affecting all organs of our body, not even sparing the eyes. The purpose of this study was to determine the pattern of ocular manifestations of HIV/AIDS and their correlation with CD4-count in a rural area of India. *Methods.* A hospital based observational cross-sectional study was done on 40 HIV-positive patients presenting to ART center with ocular complaints. Data were collected using face-to-face interview, clinical examination, slit lamp examination, fundus examination, and laboratory investigations. *Results.* Out of 40 patients, 21 were males and 19 were females with mean age of 38.75 ± 13.9 years. HIV retinopathy was the most common HIV-associated ophthalmic lesion while anterior uveitis was the most common anterior segment finding. Posterior segment lesions showed significant association (*P* < 0.05) with low CD4-count of the patient. CMV retinitis, retinal detachment, tubercular chorioretinitis, and acute retinal necrosis were all seen in patients with CD4-count less than 100 cells/mm^3^. *Conclusions.* HIV retinopathy, CMV retinitis, herpes zoster ophthalmicus, and anterior uveitis are common ocular manifestations associated with HIV infection. Low CD4-count is a risk as well as predictor for ocular manifestations. There needs to be awareness of ocular involvement among HIV infected individuals and an increased emphasis on regular ophthalmic examination.

## 1. Introduction

Since the report of an unusual occurrence of* Pneumocystis* carinii pneumonia in five cases on June 5, 1981, by Gottlieb and colleagues (*Pneumocystis* pneumonia—Los Angeles), which was probably the first publication on this infection, a great collection of literature has accumulated regarding this devastating illness [1]. In the 30 years, since the appearance of that article, human immunodeficiency virus (HIV), the causative agent of acquired immune deficiency syndrome (AIDS), has been identified and has reached virtually every corner of the globe, emerging as the most challenging pandemic of our time. It appears to be omnipresent, with the manifestations sparing no organ.

Among HIV-positive individuals, the lifetime cumulative risk for developing at least one abnormal ocular lesion ranges from 52% to 100% in various studies [2]. Such lesions are varied and affect almost any structure of the eye. Ocular lesions usually occur in the late phase of HIV infection but can also be the presenting manifestation of the disease. Various ocular manifestations—including cytomegalovirus (CMV) retinitis, toxoplasma retinochoroiditis, ocular tuberculosis, and toxoplasma retinochoroiditis—are considered to be AIDS-defining conditions.

Since it was first described in 1981, AIDS has become a major concern to all doctors, irrespective of their area of study or specialization. Even the ophthalmologists have not been spared. They sometimes make the initial diagnosis of AIDS; most often, however, they are requested to help treat the ocular manifestation related opportunistic infection. These can have disastrous consequences for sight, especially for patient who are first seen when already markedly debilitated. Ophthalmologists are faced with the challenge to recognize and treat potentially sight threatening conditions and to identify unusual presentations. They are sometimes first to diagnose the disease based on suspicious ocular clinical presentation of patients. If these ocular manifestations are detected at an early stage and treated promptly, it will be helpful to prevent or minimize consequent visual damage.

As part of our efforts to provide best of care to HIV patients, prevention of visual morbidity in these patients because of ocular complications also needs to be addressed. Information regarding these ocular manifestations is unavailable from a rural area of India. Till date, to the best of our knowledge, there has been no study indicating the ocular manifestations of HIV/AIDS from a rural area of India. Hence, this study was undertaken to identify the ocular manifestations of HIV/AIDS in a rural area of western Maharashtra in India.

## 2. Methods

### 2.1. Study Design

The present study was a hospital based observational cross-sectional study carried out at the Department of Ophthalmology of Rural Medical College and Pravara Rural Hospital of Pravara Institute of Medical Sciences, Loni, from the period of August 2011 to August 2013 (2 years). The hospital has an ART center affiliated to National AIDS Control Organisation (NACO) and catering to around 260 villages in the region.

### 2.2. Inclusion Criteria

HIV-positive patients registered at the ART centre and referred to Ophthalmology OPD for ocular complaints were included in the study. Patients presenting to ophthalmology OPD directly who were not originally known to be HIV-positive and were subsequently tested for and diagnosed to be HIV-positive because of suspicious ocular lesions were also included.

### 2.3. Data Collection

Data was collected using interview, clinical examination, and laboratory investigations. Distance and near vision were tested using Snellen's distance and Jaeger near vision chart, respectively. Anterior segment examination was done with the help of slit lamp biomicroscope. Fundus examination with indirect ophthalmoscopy was done in all patients. Positive findings were documented on Zeiss slit-lamp camera, Topcon fundus camera, and digital camera. Fundus fluorescein angiography, ultrasound examination, and other ancillary investigations such as magnetic resonance imaging and infectious agent antibody titres were obtained in cases wherever necessary. CD4-count was obtained in all cases.

### 2.4. Statistical Analysis

Mean and standard deviation were used as descriptive statistical tools and chi-square was used as inferential statistical tool. A comparison of all the ocular manifestations of adnexal, anterior segment, posterior segment, and neuroophthalmic lesions was done in relation to the CD4-count at presentation. To analyze the association we applied Chi-square test (*χ* ^2^-test) to the data wherever possible and *P* < 0.05 was considered significant.

### 2.5. Ethical Issues

This study was conducted according to the principles expressed in the Declaration of Helsinki. Ethical approval for this study was obtained from the Institutional Ethical Committee and Research Cell of Pravara Institute of Medical Sciences. A written informed consent was obtained from all patients before including them in the study and only those who consented were studied.

## 3. Results

40 HIV-positive patients with ocular complaints were included in the study. There were 21 males and 19 females. Male-to-female ratio was 1.1 : 1. Age of the patients ranged between 6 years and 63 years with a mean age of 38.75 years ± 13.9 (standard deviation) (Table 1). The median age of the 40 patients was 39.5 years. Youngest patient was 6 years old and oldest was 63 years of age.

**Figure 1:**
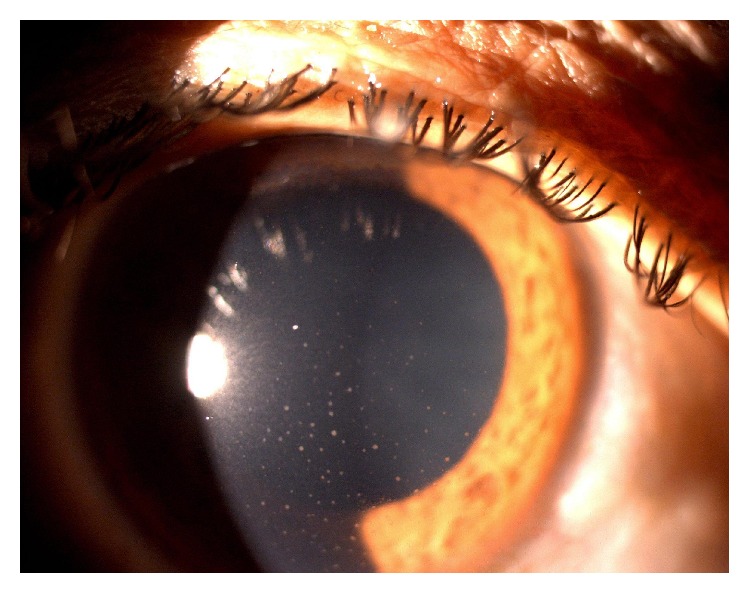
Anterior uveitis.

**Figure 2:**
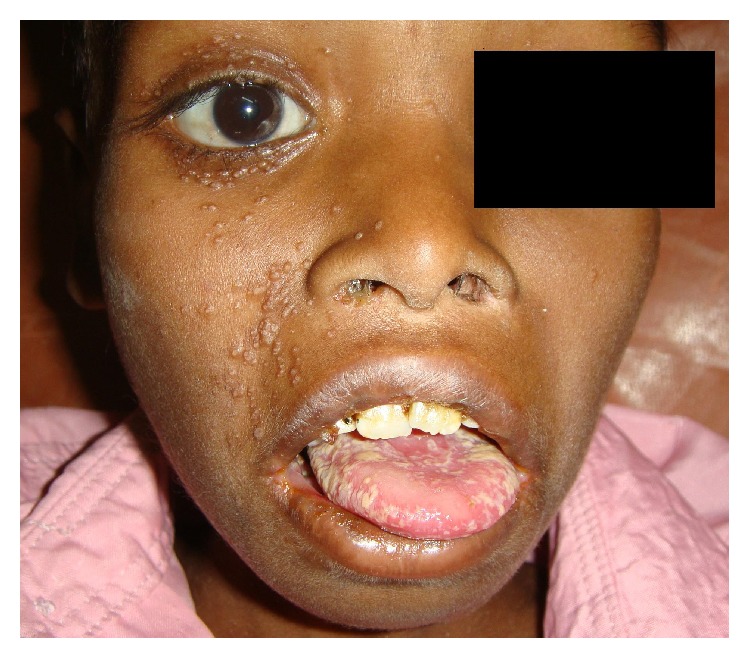
Molluscum contagiosum on right eyelids and cheek.

**Figure 3:**
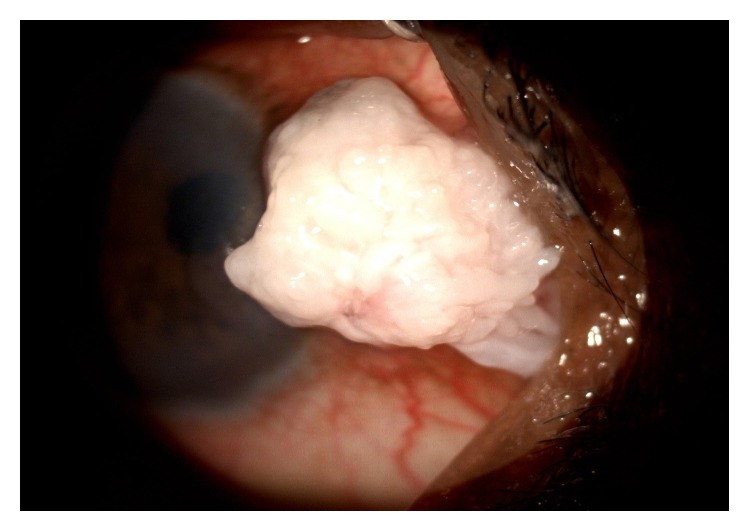
Squamous cell carcinoma of conjunctiva.

A total of 26 patients (65%) had anterior segment lesions. 27 anterior segment lesions were found among 26 patients (Table 2). The most common anterior segment finding was anterior uveitis (5 patients, 12.5%) (Figure 1) followed by molluscum contagiosum (Figure 2) and viral keratitis. Other lesions seen in the anterior segment were blepharitis, ocular surface squamous neoplasia (OSSN) (Figure 3), and Steven Johnson's syndrome (SJS).

**Figure 4:**
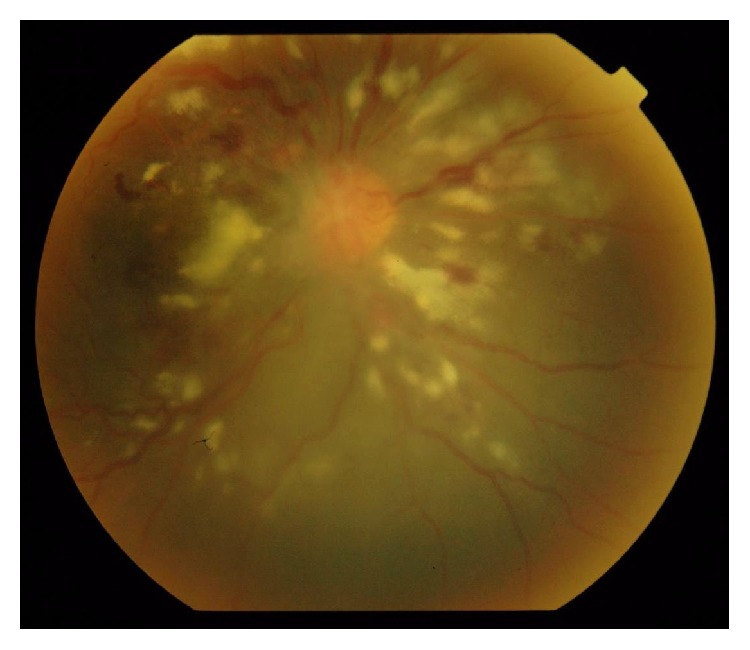
Cytomegalovirus retinitis.

**Figure 5:**
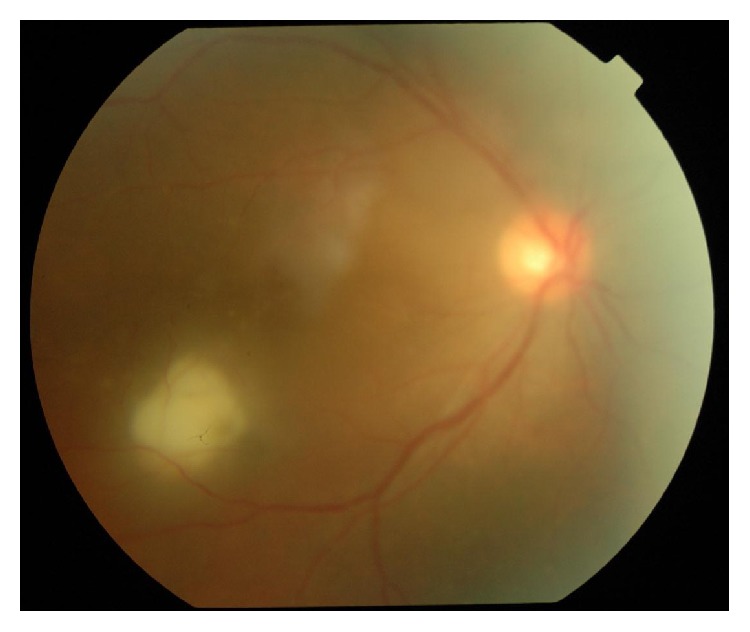
Active toxoplasmic retinochoroiditis.

**Figure 6:**
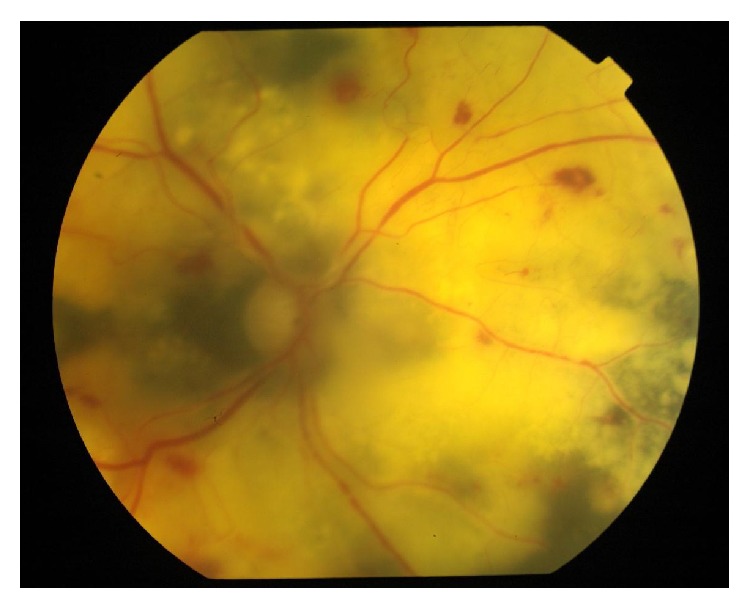
Acute retinal necrosis.

Out of 40 patients, 16 patients (40%) had posterior segment lesions. A total of 25 posterior segment lesions were found among 16 patients (Table 3). Opportunistic infections of the retina and choroid were the most common posterior segment finding seen in 11 patients. CMV retinitis (12.5%) (Figure 4) was the most common opportunistic infection of the retina/choroid followed by toxoplasmosis (Figure 5) and tuberculosis. Other lesions seen were HIV retinopathy and acute retinal necrosis (ARN) (Figure 6).

**Figure 7:**
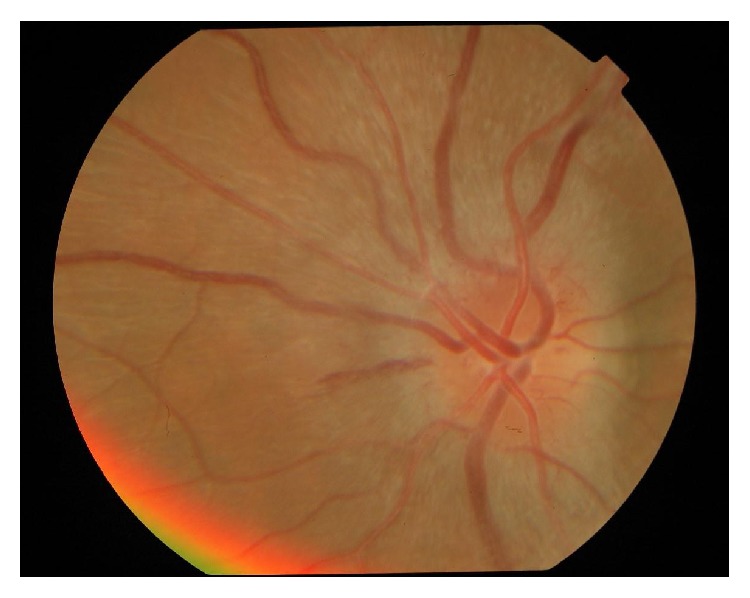
Optic neuritis in the right eye.

Table 4 enlists the neuroophthalmic lesions found in the study patients. 4 patients (10%) showed neuroophthalmic manifestations. Papilledema was the most common finding among neuroophthalmic lesions found in 2 patients. 1 patient had optic neuritis (Figure 7) while 1 patient had 3rd cranial nerve palsy.

A total of 60 eyes had some or the other ocular lesion at presentation.  Table 5 shows the best corrected visual acuity in these eyes. Based on the severity of visual impairment in the eyes with ocular lesions, the patient's eyes were divided into groups with visual acuity better than or 6/12, between 6/18 and 6/60, and worse than 6/60.

The mean CD4-counts of the patients were more of those having anterior segment lesions (mean CD4-count: 305 cells/mm^3^) than those having posterior segment lesions (mean CD4-count: 183 cells/mm^3^). None of the anterior segment lesions had mean CD4-count less than 100 cells/mm^3^. The mean CD4-count of patients with posterior segment lesions like CMV retinitis (93.8 cells/mm^3^), retinal detachment (74.7 cells/mm^3^), tuberculous chorioretinitis (96 cells/mm^3^), and ARN (64 cells/mm^3^) was less than 100 cells/mm^3^. Papilledema, the most common neuroophthalmic lesion, had mean CD4-count of 134 cells/mm^3^.

Out of total 27 anterior segment lesions, more than half (59.3%) had CD4-count of more than 200 cells/mm^3^ and 22.2% lesions had CD4-count between 100 and 200 cells/mm^3^ while 18.5% had CD4-count less than 100 cells/mm^3^ (Table 6). By applying chi-square test, no significant association was found between the anterior segment ocular lesions and low CD4-count (CD4-count less than 100 cells/mm^3^) of patients (*P* > 0.05). Out of total 25 posterior segment lesions, 44% of lesions were found in patients with CD4-count less than 100 cells/mm^3^ while 28% of lesions were found in patients with CD4-count between 100 and 200 cells/mm^3^ and more than 200 cell/mm^3^ each. There is a significant association between posterior segment ocular lesions and CD4-count (*P* < 0.05). No significant association was found between neuroophthalmic lesions and CD4-count of patients (*P* > 0.05).

## 4. Discussion

A total of 40 HIV-positive patients reporting ocular complaints were recruited in the study and examined. Male-to-female ratio was 1.1 : 1 with males comprising 52.5% of cases which is less than 61% reflected from the national statistics of HIV population [3]. Studies related to ocular manifestations of HIV carried out at various other centers like those done by Ebana Mvogo et al. and Assefa et al. also showed slight male preponderance as seen in our study [4, 5].

In our study, 42.5% of patients were in the age group of 21–40 years which is less than 55% recorded by Biswas et al. in the published series of 100 HIV-positive patients with ocular manifestations from India [6]. This can be explained by the absence of HAART at the time the latter study was conducted and hence shorter life span of the patients then. The percentage distribution of patients according to age in our study was 10% in pediatric age group (up to 12 years of age), 72.5% among adults (20 to 50 years of age), and 17.5% among those above 50 years of age. This is also seen in the national statistics of HIV-infected population (4% among children below the age of 15 years, 83% among adults, and 13% among those aged over 50 years) [3]. In the present study, 75% belonged to the economically productive age group of 20–50 years. This needs emphasis as the morbidity of these patients has a considerable impact on the economy of their family.

### 4.1. Anterior Segment Findings

In our study, 65% of cases had anterior segment manifestations. Similar percentage of anterior segment involvement cases were reported by Assefa et al. [5].

Anterior uveitis (12.5%) was the most common anterior segment finding. Sudharshan et al. in their recent work of ocular lesions in 1000 consecutive HIV-positive patients in India found anterior uveitis in 16% of the patients [7]. The most likely cause of anterior uveitis was determined clinically. 2 cases of anterior uveitis were concurrent with CMV retinitis which was most probably because of the immune recovery rather than the CMV disease process itself as anterior uveitis is very rare because of CMV retinitis. 1 patient had “spill-over” anterior uveitis subsequent to toxoplasma retinochoroiditis. Another patient developed immune recovery uveitis characterized by severe anterior uveitis and vitritis along with marked diminution of vision. She was recently started (3 months back) on HAART when her CD4-count was 215 cells/mm^3^ and had now improved to 496 cells/mm^3^.

Molluscum contagiosum affects up to 5% of the HIV-infected patients [8]. Children account for 90% of molluscum contagiosum episodes [9]. Out of the 4 patients with molluscum contagiosum in our study, 2 were of pediatric age group. A cross-sectional hospital based study in Uganda showed that over 10% of pediatric HIV patients had molluscum contagiosum [10].

Herpes zoster ophthalmicus (HZO) and viral keratitis were found in 5% and 10% patients, respectively. This was similar to findings reported by Ndoye et al. and Ebana Mvogo et al. [4, 11]. However Biswas et al. and Msosa and Kollmann reported the prevalence of HZO as 0.8% and 1%, respectively [6, 12]. However, it is difficult to draw firm conclusions about these differences as study populations have differed with regard to levels of immunodeficiency, ART exposure, and methods of case ascertainment.

OSSN was found in 2 (5%) of the study patients and both of them were below the age of 50 years. HIV-induced immunosuppression resulting in reduction in the effectiveness of the immune surveillance system has already been proven to be a risk factor for squamous cell carcinoma of the conjunctiva in a case control study in Rwanda by Kestelyn et al. [13]. There was not even a single case of Kaposi's sarcoma in our study as was the case in many other Indian studies. This may possibly be due to the rarity of the human herpes virus 8 in the Indian subcontinent and human herpes virus 8 has been implicated in the causation of Kaposi's sarcoma [14, 15].

### 4.2. Posterior Segment Findings

Posterior segment lesions were seen in 24 patients (60%). The most common posterior segment lesion was HIV retinopathy in 17.5% of patients. This finding correlates well with the study by Biswas et al. (12.8%) in India but is quite less than compared to similar studies in USA (Holland et al., 53%, and Kuppermann et al., 45%) and Africa (Kestelyn et al., 30%) [6, 13, 16, 17]. In our series, majority of the patients were referred due to visual impairment. As HIV retinopathy alone does not cause much visual impairment, they probably remained undetected.

The most common ophthalmic opportunistic infection seen in the study was CMV retinitis (12.5%). This is similar to frequency of CMV retinitis reported in other studies from India like those done by Gharai et al. (20%), Pathai et al. (11.9%), and Biswas et al. (17%) [6, 18, 19]. However studies from Africa have reported very less frequency of CMV retinitis (around 1% or less) [5, 10, 11]. The low prevalence of CMV retinitis in countries of Africa as compared to America and Europe may not be a direct reflection of lower incidence but possibly reflects that the patients die from systemic opportunistic infections before their CD4-count falls low enough to allow development of CMV retinitis.

Ocular toxoplasmosis was the next most common ocular opportunistic infection and was seen in 3 patients (7.5%). Sudharshan et al. in their study on 1000 consecutive HIV-positive patients in India found the frequency of ocular toxoplasmosis to be 4.1% which is similar to that found in our study [7].

Ocular tuberculosis was seen in 2.5% patients. It presented as tuberculoma and disseminated choroiditis. Although systemic tuberculosis was the commonest underlying systemic infection, ocular TB was relatively rare. The low frequency of ocular tuberculosis in our study may be due to the inclusion of HIV-positive patients with ocular complaints as asymptomatic choroidal tubercles are among the commonest manifestations of ocular TB in HIV.

Two patients (5%) had retinal vascular occlusions. One patient (2.5%) presented with central retinal artery occlusion along with 3rd cranial nerve palsy. Cases have been reported in literature of isolated cranial nerve palsy and vascular occlusions in HIV patients but are relatively rare, arterial occlusions being very rare [20, 21].

One patient presented with anterior uveitis in both eyes and fundus showed large granular areas of retinal whitening along with vitritis and generalized retinal vasculitis suggestive of ARN. Pfaffl et al. reported ARN in 4 patients out of 538 HIV-positive patients they followed up for 5 years [22]. Batisse et al. inferred from their study of 26 patients of ARN in HIV-positive individuals that ARN is a late event in the course of immunosuppression (CD4-count < 100 cells/mm^3^) [23]. This was also highlighted in our patient whose CD4-count was 64 cells/mm^3^ and duration since diagnosis of HIV was 8 years.

### 4.3. Neuroophthalmic Lesions

In the study, 10% (4 patients) of total cases had neuroophthalmic lesions. This frequency of neuroophthalmic manifestations was similar to that reported by Assefa et al. (9.6%), Sudharshan et al. (8.9%), and Biswas et al. (9.3%) [5, 7, 24].

### 4.4. CD4-Count Association

There was no significant association between anterior segment lesions and CD4-count of the patients. In general, the CD4-count in majority of cases (59.3%) of anterior segment lesions was more than 200 cells/mm^3^. Few patients of anterior segment lesions which had CD4-count less than 100 cells/mm^3^ also had concurrent posterior segment lesion. The CD4-count distribution of various adnexal and anterior segment lesions is nearly similar to earlier studies by Ndoye et al., Cunningham Jr. and Margolis, and Bekele et al. [11, 25, 26].

Posterior segment lesions showed significant association with low CD4-count. Most of the posterior segment lesions (72%) had CD4-count less than 200 cells/mm^3^. All the active cases of CMV retinitis had CD4-count less than 100 cells/mm^3^ as already highlighted by Jabs et al. in their study way back in 1989 [27]. 66.7% patients of toxoplasma retinochoroiditis had CD4-count between 101 and 200 cells/mm^3^. This finding shows that opportunistic ocular infection due to toxoplasma gondii increases as immunity of patient decreases, especially when CD4-cell count becomes less than 200 cell/mm^3^ as reported in earlier studies [28]. All cases of retinal detachment had CD4-count less than 100 cell/mm^3^. This may be because retinal detachment was seen as complication of CMV retinitis and tuberculous retinochoroiditis which generally occur in low CD4-count. There was no significant association between neuroophthalmic lesions and CD4-count of the patient. Like in studies by Bekele et al. and Kumar et al., in our study too neuroophthalmic lesions were seen in the entire spectrum of CD4-count [26, 29].

## 5. Conclusions

Ocular manifestations in HIV/AIDS affect mainly the economically productive and socially important age group of 20 to 50 years with slight male preponderance. HIV retinopathy was the most common ocular lesion encountered in our study with mild visual deterioration and good immune status. CMV retinitis was the next common lesion with severe degree of visual impairment and poor immune status. Anterior uveitis was the most common anterior segment manifestation. Systemic tuberculosis was the most common coexistent systemic disease in our study. The percentage of posterior segment ocular manifestations of HIV increased as the CD4-count of HIV-positive patients decreased. The severity of ocular manifestations in HIV/AIDS with respect to visual impairment was higher in patients with low CD4-counts. Hence the patients with serological diagnosis of HIV should be examined for ocular involvement and correlated to CD4-count for treatment and better visual prognosis.

## Figures and Tables

**Table 1:** Age-wise distribution of patients.

Age (years)	Number of patients
0–10	3 (7.5%)
11–20	1 (2.5%)
21–30	5 (12.5%)
31–40	12 (30%)
41–50	12 (30%)
51–60	5 (12.5%)
61–70	2 (5%)
Total	**40**
Mean age	**38.75 ± 13.9 years**

**Table 2:** Adnexal and anterior segment lesions.

Anterior segment lesions	Number of patients	Percentage of total patients (*n* = 40)	Percentage of adnexal/anterior segment lesions (*n* = 27)
Anterior uveitis	5	12.5	18.5
Molluscum contagiosum	4	10	14.8
Viral keratitis	4	10	14.8
Blepharitis	4	10	14.8
Herpes zoster ophthalmicus	2	5	7.4
Dry eyes	3	7.5	11.1
Giant papillary conjunctivitis	2	5	7.4
Ocular surface squamous neoplasia	2	5	7.4
Steven Johnson's syndrome	1	2.5	3.7

**Table 3:** Posterior segment lesions.

Posterior segment lesions	Number of patients	Percentage of total patients (*n* = 40)	Percentage of posterior segment lesions (*n* = 25)
HIV retinopathy	7	17.5	28
CMV retinitis	5	12.5	20
Toxoplasma retinochoroiditis	3	7.5	12
Retinal detachment	3	7.5	12
Retinal vascular occlusions	2	5	8
Retinopathy of anaemia	2	5	8
Tubercular chorioretinitis	1	2.5	4
Acute retinal necrosis	1	2.5	4
Endogenous endophthalmitis	1	2.5	4

**Table 4:** Neuroophthalmic lesions.

Neuroophthalmic lesions	Number of patients	Percentage of total patients (*n* = 40)	Percentage of neuroophthalmic lesions (*n* = 4)
Papilledema	2	5	50
Optic neuritis	1	2.5	25
3rd cranial nerve palsy	1	2.5	25

**Table 5:** Visual impairment in eyes with ocular lesion/s (*n* = 60).

Visual acuity	Segment of eye involved				Total
	Only adnexal and anterior segment	Only posterior segment	Only neuroophthalmic lesions	Combination of adnexal/anterior, posterior, and neuroophthalmic lesions	
Better than or 6/12	22 (36.7%)	3 (5%)	2 (3.3%)	3 (5%)	**30 (50%)**
6/18 to 6/60	6 (10%)	7 (11.7%)	2 (3.3%)	—	**15 (25%)**
Worse than 6/60	1 (1.7%)	6 (10%)	1 (1.7%)	7 (11.7%)	**15 (25%)**
Total	**29** **(48.3%)**	**16** **(26.7%)**	**5** **(8.3%)**	**10** **(16.7%)**	**60**

**Table 6:** Association between ocular lesions and CD4-count.

Ocular lesions	CD4-count (cells/mm^3^)			Total
	<100	100–200	>200	
Anterior segment (*n* = 27)				
Anterior uveitis	2 (7.4%)	2 (7.4%)	1 (3.7%)	**5 (18.5%)**
Molluscum contagiosum	2 (7.4%)	—	2 (7.4%)	**4 (14.8%)**
Viral keratitis	—	1 (3.7%)	3 (11.1%)	**4 (14.8%)**
Blepharitis	1 (3.7%)	—	3 (11.1%)	**4 (14.8%)**
Herpes zoster Ophthalmicus	—	1 (3.7%)	1 (3.7%)	**2 (7.4%)**
Dry eye	—	1 (3.7%)	2 (7.4%)	**3 (11.1%)**
Giant papillary Conjunctivitis	—	—	2 (7.4%)	**2 (7.4%)**
OSSN	—	—	2 (7.4%)	**2 (7.4%)**
SJS	—	1 (3.7%)	—	**1 (3.7%)**
Total	**5 (18.5%)**	**6 (22.2%)**	**16 (59.3%)**	**27**

Posterior segment (*n* = 25)				
HIV retinopathy	2 (8%)	2 (8%)	3 (12%)	**7 (28%)**
CMV retinitis	4 (16%)	1 (4%)	—	**5 (20%)**
Toxoplasma retinochoroiditis	—	2 (8%)	1 (4%)	**3 (12%)**
Retinal detachment	3 (12%)	—	—	**3 (12%)**
Retinal vascular occlusions	—	1 (4%)	1 (4%)	**2 (8%)**
Retinopathy of anaemia	—	1 (4%)	1 (4%)	**2 (8%)**
Tubercular chorioretinitis	1 (4%)	—	—	**1 (4%)**
ARN	1 (4%)	—	—	**1 (4%)**
Endogenous endophthalmitis	—	—	1 (4%)	**1 (4%)**
Total	**11 (44%)**	**7 (28%)**	**7 (28%)**	**25**

Neuroophthalmic (*n* = 4)				
Papilledema	1 (25%)	—	1 (25%)	**2 (50%)**
Optic neuritis	—	—	1 (25%)	**1 (25%)**
3rd cranial nerve palsy	—	—	1 (25%)	**1 (25%)**
Total	**1 (25%)**	**0**	**3 (75%)**	**4**

## Abbreviations

AIDS: Acquired immunodeficiency syndrome
ARN: Acute retinal necrosis
ART: Antiretroviral therapy
CMV: Cytomegalovirus
DNA: Deoxyribonucleic acid
HAART: Highly active antiretroviral therapy
HIV: Human immunodeficiency virus
HZO: Herpes zoster ophthalmicus
MRI: Magnetic resonance imaging
NACO: National AIDS Control Organisation
PCR: Polymerase chain reaction
TB: Tuberculosis.
